# Methodology for biomechanical investigation of implant malpositioning in total knee arthroplasty using a six degree of freedom joint simulator

**DOI:** 10.1186/s42836-025-00351-w

**Published:** 2025-12-09

**Authors:** Eric Kleist, Paul Henke, Christoph Woernle, Rainer Bader, Maeruan Kebbach, János Zierath

**Affiliations:** 1 Chair of Technical Mechanics/Dynamics, University of Rostock, 18051 Rostock, Germany; 2 Biomechanics and Implant Technology Research Laboratory, Department of Orthopedics, Rostock University Medical Center, 18057 Rostock, Germany

**Keywords:** Biomechanics, Joint simulator, Total knee arthroplasty, Implant positioning, Joint dynamics, Multibody model

## Abstract

The implantation of total knee replacements is an effective treatment for advanced degenerative knee joint diseases. Implant positioning relative to the bones affects the loads occurring in the artificial joint, joint stability, and postoperative functionality. Variance in implant positioning during the surgical implantation of a total knee replacement cannot be entirely ruled out. By simulating implant malpositioning in an experimental setting, particularly critical cases of malalignment can be identified, from which guidelines for orthopedic surgeons can be derived. The AMTI VIVO™ six degree of freedom joint simulator allows reproducible preclinical testing of joint endoprostheses under specific kinematic and loading conditions. It features a virtual ligament model that defines up to 100 ligament fibers between the articulating components. This paper presents a method to investigate the effect of different implant positions on the biomechanics of the knee after total knee arthroplasty. For this, the VIVO joint simulator requires no modification of the physical setup by moving virtual ligament insertion points relative to the bone. As a proof of concept, exemplary shifts and rotations of the femoral and tibial implant components are performed, and dynamic results are compared to a musculoskeletal multibody digital twin and findings from the literature.

Video Abstract

**Supplementary Information:**

The online version contains supplementary material available at 10.1186/s42836-025-00351-w.

## Introduction

The human knee joint is one of the largest, most heavily loaded [1], and most injury-prone [2] joints. An effective treatment for advanced degenerative diseases of the knee joint is the implantation of total knee endoprostheses [3–6]. In 2023, total knee arthroplasty (TKA) was performed approximately 230,000 times in Germany, making it the 8th most common surgical procedure [7]. A continuous increase in the number of procedures is expected in the future [8].

The positioning of implant components relative to the bones affects the loads occurring in the artificial joint, joint stability, and postoperative functionality. Implant positioning thus has an impact on long-term treatment success and patient satisfaction [9–11]. However, variance in implant positioning during the surgical implantation of a total knee replacement (TKR) cannot be entirely ruled out. For instance, Siston et al. [12] reported postoperative deviations in the alignment of the femoral implant of up to 13° internally or 16° externally from its target position. By simulating implant malpositioning in the bone in an experimental setting, particularly critical cases of misalignment can be identified, from which guidelines for orthopedic surgeons can be derived.

The analysis of the influence of implant positioning has already been the subject of numerous studies [13–29]. While adjusting implant positions is typically straightforward in numerical simulation models, experimental setups often require modifications to physical components. For example, custom-made implants with altered geometries may be used to achieve the desired changes [21, 22]; however, not all research groups have access to such resources.

In this context, the AMTI (Advanced Mechanical Technology, Inc., Watertown, MA, USA) VIVO™ joint simulator (hereafter referred to as “VIVO joint simulator”) used in this investigation is capable of moving two joint components with six relative degrees of freedom (DOF) using two independently movable manipulators. It can be used to study various joint implants, including knee, hip, or shoulder replacements. The VIVO joint simulator also features a virtual ligament model that allows for the definition of up to 100 ligament fibers between the articulating components. This enables joints to be examined under realistic conditions without the need for a physical joint capsule or ligaments.

The simulation of implant malalignment using a VIVO joint simulator was previously investigated [27–29]. The first study [27] does not elaborate on the specific methods used to achieve implant position variation. The other studies achieved variations either by modifying physical components [28] or by using an additional commercially available extension software (VIVO Sim Visualization Software, AMTI, Watertown, MA, USA), which provided a digital twin of the simulated joint alongside the VIVO joint simulator [29]. This present study describes a method for varying implant positions on the VIVO joint simulator entirely virtually and without the need for this extension software. This is achieved by moving virtual ligament insertion points relative to the bone. The method requires no modification of physical components and theoretically allows for implant position variation in any direction and of any magnitude. As a proof of concept, exemplary shifts and rotations of the femoral and tibial implant components are performed. The results may serve as a supplement to the existing literature.

## Materials and methods

The following sections first describe the numerical simulation setup of the human lower extremity. It was developed to serve as a digital twin of the experimental setup presented afterwards, featuring a six DOF joint simulator.

### Numerical simulation setup

**Fig. 1:**
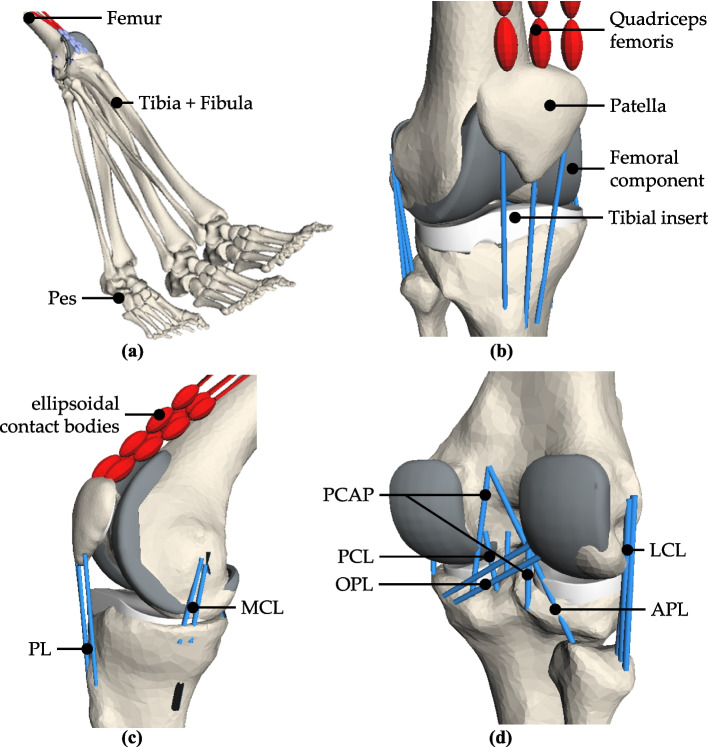
Musculoskeletal multibody system of the lower right limb with relevant bones, implants, ligaments (blue), and muscles (red). **a** Whole MMBS in different flexion positions. **b** Anterior-lateral view of the knee joint. **c** Medial view. **d** Posterior-lateral view

As a reference for validating experimental results obtained with the VIVO joint simulator, a musculoskeletal multibody system (MMBS) model of the human lower extremity was built up using the multibody simulation software SIMPACK (Version 2022x, Dassault Systèmes, France). The model includes the bony structures of the lower right limb (pelvis, femur, tibia and fibula, patella, pes) of a male cadaver specimen (age 74 years, height 176 cm, weight 80 kg) and the implants (femoral implant component, tibial insert, patellar component) represented as 3D surface geometries in STL format (Fig. 1a and b).

A bicondylar, posterior cruciate ligament (PCL)-retaining, fixed bearing TKR design (Multigen Plus Knee System, Size 3, Lima Corporate, Italy) was used. The ultra-high molecular weight polyethylene patellar component (also part of the Multigen Plus system; diameter 32 mm, height 8 mm) was positioned on the posterior side of the patella.

The virtual positioning of the implants within the bones was performed under the guidance of an experienced orthopedic surgeon. Two body-fixed prosthesis coordinate systems *K*_f_ (femoral implant component) and *K*_t_ (tibial insert), were defined as a reference for evaluating joint kinematics. The axes and origin points of these coordinate systems were defined according to ISO 14243–1 [30] as well as recommendations from the VIVO’s manufacturer [31].

The MMBS was modeled as an open kinematic chain consisting of rigid bodies with the pelvis and femur being fixed and the patella, tibia/fibula, and pes being movable. A polygonal contact model was defined between the articulating surfaces of the implant components [41]. The MMBS mimicked the clinically relevant load case of a passive knee flexion from 0° to 80° in a seated position without contact of the foot to the ground, as shown in Fig. 1a. For this purpose, the tibiofemoral joint was modeled with five DOF, while the flexion angle was predefined as a rheonomic constraint. The patella was modeled with six DOF. The simulation output provides the remaining joint kinematics and dynamics of the tibia and patella, derived from the equilibrium conditions between joint contact-, quadriceps- and ligament forces. The influence of gravity was neglected for the simulation.

Additionally, the anatomical surroundings of the joint were modeled, including ligaments and a selection of muscles (Fig. 1b–d). The following tibiofemoral ligaments were included in the MMBS model: two bundles of the PCL; three bundles each of the medial and lateral collateral ligament (MCL/LCL); one bundle of the oblique posterior medial collateral ligament (opMCL); two bundles of the deep medial collateral ligament (dMCL); two bundles of the oblique popliteal ligament (OPL); one bundle of the arcuate popliteal ligament (APL); and one bundle each of medial and lateral posterior capsule (mPCAP, lPCAP). The individual ligament forces *F* are calculated using a non-linear force-strain law introduced by Wismans [32]:

1 $$F\left(\varepsilon \right)=\left\{\begin{array}{c}0\\ \frac{K}{{4}\ {\varepsilon _1}}{\varepsilon }^{2}\\ K\left(\varepsilon -\varepsilon _1\right)\end{array}\begin{array}{l}\text{for} \ \varepsilon \le 0\left(\text{ligament unstrained}\right)\\ \text{for} \ 0<\varepsilon \le {2}\ \varepsilon _1 \left(\text{quadratic section}\right)\\ \text{for} \ \varepsilon>{2}\ {\varepsilon _1}\left(\text{linear section}\right)\end{array}\right.$$

with *K* representing the ligament stiffness value, *ε* being the current ligament strain, and *ε*_1_ representing the transition value between quadratic and linear behavior. Mechanical parameters and ligament insertion point locations were adopted from the literature [33] and partially modified to be compatible with the virtual ligament model of the joint simulator described in the *Description of the joint simulator* Section. Table 1 provides an overview of all ligaments considered, including their stiffness values used in the underlying force law (Eq. 1) and their strains in a reference configuration, defined as the equilibrium state of the knee joint at 0° flexion. Each ligament has a transition value *ε*_1_ of 0.03 (corresponding to a transition strain of 6% according to Eq. 1). Due to its high stiffness, the patellar ligament (PL) was modeled as a non-elastic coupling element between the distal apex of the patella and the tibial tuberosity [34, 35].

**Table 1:** Considered ligaments, stiffnesses, and reference strains in the musculoskeletal model

**Ligament**	**Bundle** ^1^	**Stiffness**	**Ref. strain**	**Ligament**	**Bundle** ^1^	**Stiffness**	**Ref. strain**
PCL	a	6468.8	−0.51	LCL	a	1209.1	0.12
	po	2101.2	−0.33		s	3274.7	0.07
MCL	a	2388.1	0.07		po	3273.9	0.11
	c	3355.4	0.06	OPL	pr	917.3	0.10
	po	3595.5	0.06		d	871.0	0.11
opMCL	-	2700.0	−0.01	APL	-	2071.9	0.07
dMCL	a	3000.0	−0.09	PCAP	l	5264.3	0.06
	po	4000.0	0.02		m	5069.7	0.06
PL	non-elastic coupling element						

^1^a: anterior, c: central, d: distal, l: lateral, m: medial, po: posterior, pr: proximal, s: superior

The M. quadriceps femoris was also modeled, divided into four muscle bundles: M. rectus femoris, M. vastus medialis, M. vastus lateralis, and M. vastus intermedius. A total force of 40 N was distributed among the muscle bundles to keep the patella in position. Similar quadriceps loads have been used in comparable studies investigating passive movements or static load cases in the knee joint [36–40]. Wrapping of the M. quadriceps femoris around the Facies patellaris of the femoral implant component was simulated by defining a polygonal contact between the implant surface and a chain of ellipsoidal bodies integrated into the distal portions of the muscle strands (Fig. 1b and 1c) [26, 41, 42].

### Experimental setup

#### Description of the joint simulator

The VIVO joint simulator used in this investigation enables performance analyses of various types of joint implants under physiological loading conditions. A detailed description of the VIVO joint simulator’s hardware and software was provided in a previous publication [43]. The following section offers an overview of its key functionalities used in the present study.

**Fig. 2:**
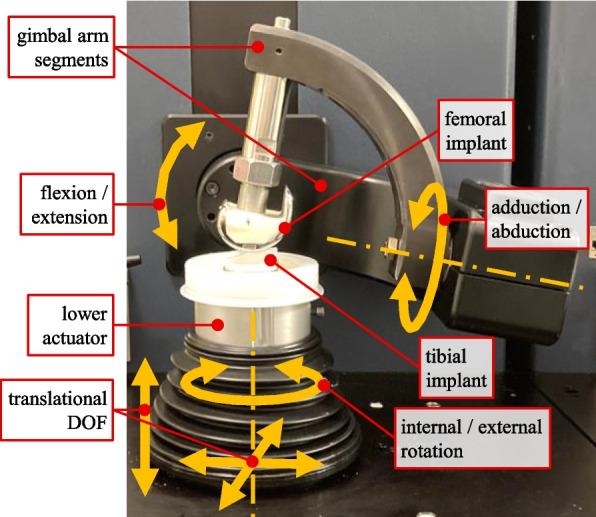
VIVO joint simulator with mounted femoral and tibial knee implants and schematic depiction of DOF. Upper actuator (gimbal arm segments) with two rotational DOFs for flexion/extension and adduction/abduction; lower actuator providing omnidirectional translations and internal/external rotation

Equipped with two servo-hydraulically driven actuators, the VIVO joint simulator allows relative motion between two articulating implant components with up to six DOF (Fig. 2). It features a virtual ligament model capable of simulating up to 100 ligament fibers, each acting as a one-dimensional force element. Virtual ligament forces act along straight lines between insertion points defined in the upper and lower actuator coordinate systems, respectively. The force calculation follows the same non-linear force law applied in the MMBS (Eq. 1).

The simulator can be operated using either a proportional-integral (PI) controller or through an iterative learning control (ILC) algorithm provided by the manufacturer. When using ILC, a new compensation profile is generated every third load cycle, allowing for continuous reduction of tracking errors. ILC was applied in the investigations conducted for this study.

#### Integration of the TKR into the experimental setup

**Fig. 3:**
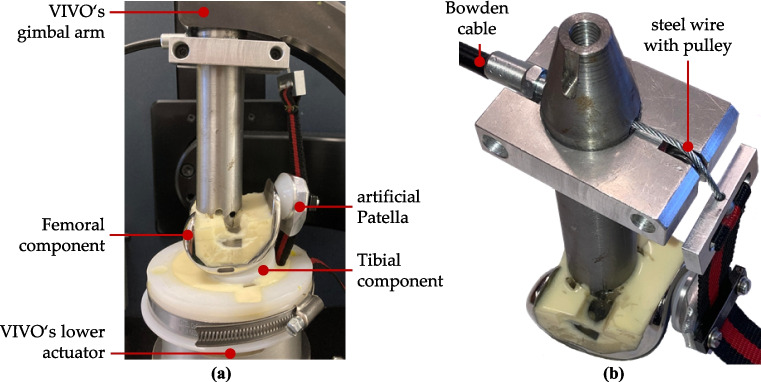
Integration of the prosthesis components into the VIVO joint simulator. **a** Complete TKR system within the VIVO joint simulator. **b** Detailed view of the guide for the quadriceps wire

The same TKR design that was modeled in the MMBS was physically integrated into the VIVO joint simulator (Fig. 3a). The femoral prosthesis component was mounted on the upper gimbal arms of the VIVO joint simulator, and the tibial insert was fixed on the lower cross table. To verify implant positioning after embedding, a geometric measurement method was applied using a coordinate measuring device [44]. The patellar component was attached to a custom aluminum part designed to replicate the shape and dimensions of the human patella, including key anatomical landmarks such as the distal apex patellae. This setup ensures that the forces exerted by the M. quadriceps femoris and the PL on the patella are as physiologically as possible. A clamping mechanism on the anterior surface of the patellar mount allows attachment of the artificial M. quadriceps femoris and PL. These structures were modeled using a low-stretch polypropylene strap, which supports high load application (up to 1250 N) and, due to its 25 mm width, approximates the anatomical cross-sectional extent of the muscle and ligament. Additionally, the soft strap can wrap around the femoral prosthesis component without damaging the artificial facies patellaris. Proximally, the quadriceps strap is connected to a flexible steel wire, which is routed out of the simulation environment via a Bowden cable (Fig. 3b) and can be loaded at its free end.

Superior to the patella, the M. quadriceps femoris is anatomically composed of several individual muscle strands, which proximally insert at various locations on both the femur and the pelvis. These individual lines of action were not explicitly modeled in the experimental setup. Instead, the penetration points of the individual muscles in a transverse plane of the femur, located approximately 150 mm superior to the joint in an extended position, were determined using the MMBS. The geometric center of these points was then defined as the reference point for applying the M. quadriceps femoris force.

The distal insertion of the PL on the tibia is located at the tibial tuberosity, which lies approximately 50 mm inferior to the tibial plateau. As no actual bony structures are present in the experimental setup, the distal end of the PL was clamped to the mounting part for the tibial insert at the respective location.

#### Simulated motion and loading conditions

The simulated motion was a passive flexion motion as previously described in *Numerical simulation setup* Section. The same tibiofemoral ligaments implemented in the MMBS were replicated using the VIVO joint simulator’s virtual ligament model with identical parametrizations (stiffnesses, reference strains, and insertion points). The M. quadriceps femoris was loaded via a weight connected to the end of the steel wire, generating a constant force of 40 N.

Before conducting the experiments, a reference configuration between the implant components was established. The reference configuration was defined as the equilibrium configuration of the knee joint at 0° of flexion under ligament tension. To reduce the effects of a previously detected structural compliance of the VIVO joint simulator’s actuators on experimental outcomes, an initial vertical contact force was applied to establish the reference configuration [43]. This force was defined as the mean of the maximum and minimum forces calculated for the specific load case in the MMBS. All tests were conducted under lubricated conditions, with silicone oil (viscosity 360 mm^2^s^−1^) applied to all tibiofemoral and patellofemoral contact surfaces. A total of 300 load cycles were executed, which proved sufficient for the VIVO joint simulator’s ILC algorithm to reduce control deviations to a satisfactory level for the examined load case [43, 45].

### Implant position variations

**Fig. 4:**
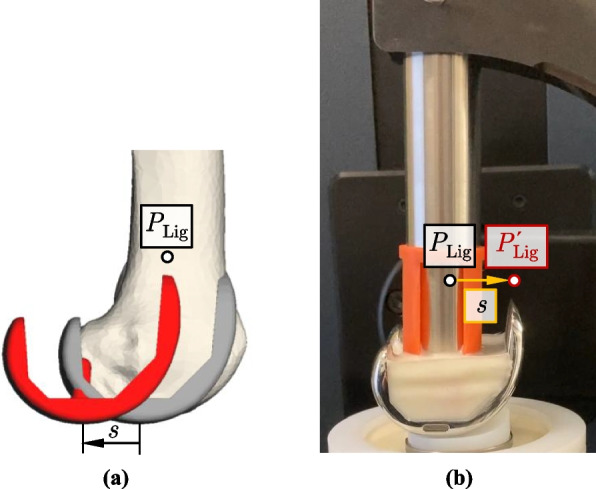
Method to simulate implant malpositioning illustrated by the posterior shift of the femoral implant component. **a** Implant shift in the body—the implant is shifted by a distance *s* while the ligament insertion points remain unchanged. **b** Simulated implant shift on the VIVO joint simulator—the insertion points are shifted by the same distance *s* in the opposite direction while the implant position remains unchanged

To consider different implant positions in the test setup, the femoral or tibial ligament insertion point clusters were moved in a specific direction. Figure 4 illustrates the methodology using an exemplary posterior shift of the femoral implant component by a deliberately large distance *s* for demonstration purposes. In the human body, the bony structures and thus the ligament insertion points (represented exemplarily by *P*_Lig_) remain in their original positions, while the implant is shifted (Fig. 4a). In the experimental setup, however, the embedded femoral implant remains in the same reference configuration relative to the joint simulator as previously defined. Instead, the insertion points are shifted by the same distance *s* in the opposite direction (in this example, anteriorly) (Fig. 4b). After performing both variants, *P*_Lig_ is located in the same position relative to the femoral implant.

A simulated shift of the tibial insert is carried out analogously to the procedure used for the femoral implant component, but by shifting the tibial insertion points. The shift and the reading of the adjusted point coordinates are performed using the MMBS. Since moving the insertion points alters ligament lengths in the reference configuration *l*_ref_, it was necessary to recalculate the individual reference strains *ε*_ref_ according to

2 $${\varepsilon}_{\text{ref}}=\frac{{l}_{\text{ref}}-{l}_{0}}{{l}_{0}}{\text{,}}$$

where *l*_0_ is the zero-strain length of the respective ligament. The adjusted insertion point coordinates and reference strains are then passed to the virtual ligament model as new parameters for each implant position variation. Apart from that, the preparation of the experiments on the joint simulator (setting the reference configuration, applying the initial compressive force) can largely be carried out as described in the *Simulated motion and loading conditions* Section. In cases where a specific implant position variation caused a considerable change in the minimum or maximum joint contact force, the initial contact force was adjusted accordingly.

**Fig. 5:**
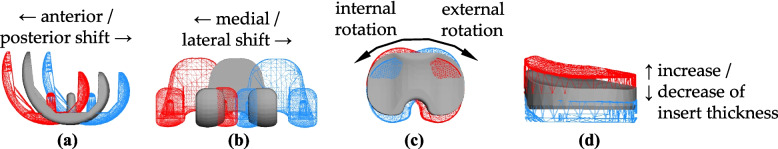
Considered variations of implant positions relative to the bone. **a** Anteroposterior shift of femoral implant. **b** Mediolateral shift of femoral implant. **c** Internal/external twist of tibial insert. **d** Increase/decrease of tibial insert thickness

The following variations in the positioning of the femoral implant component and tibial insert were investigated (Fig. 5): anterior/posterior shift of the femoral implant by ± 3 mm (Fig. 5a), medial/lateral shift of the femoral implant by ± 3 mm and ± 6 mm (Fig. 5b), twist of the tibial insert around the tibial longitudinal axis by ± 3° and ± 6° (Fig. 5c), and variation in tibial insert thickness simulated by moving the insert superiorly/inferiorly by ± 1 mm and ± 2 mm (Fig. 5d).

The implant position variations investigated represent only a subset of the possible variations; in principle, a variation of each implant component within the bone with six DOF can be simulated. The selected variations were based on those commonly examined in comparable studies [15, 17, 19, 21–27].

## Results

To verify the effectiveness of the proposed methodology for the variation of implant positions at kinematic and dynamic levels, the tibiofemoral contact force profiles along the tibial axial direction and the anteroposterior position of the femur relative to the tibia are evaluated as representative examples. Additional kinematic profiles are also analyzed for selected load cases in which considerable changes are anticipated. Translational kinematic results are reported as the shifts of the origin points of either the femur-fixed (*K*_f_) or tibia-fixed (*K*_t_) coordinate systems, relative to the origin of the other.

### Anteroposterior shift of the femoral implant component

**Fig. 6:**
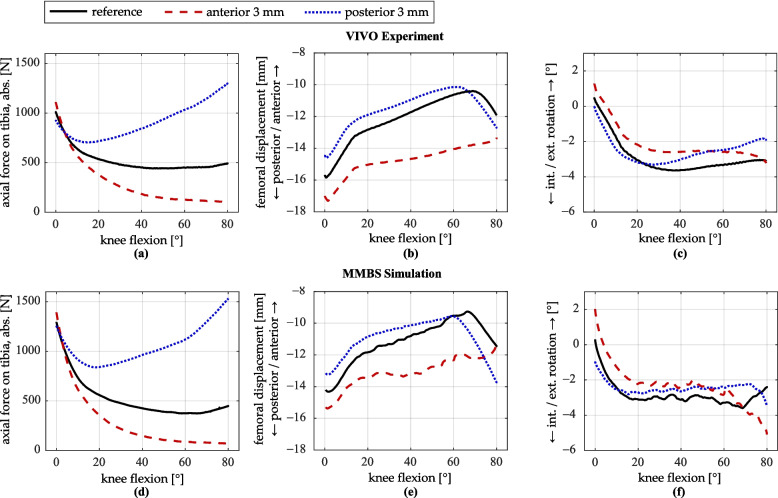
Simulation results for anteroposterior shift of the femoral implant. **a**–**c** VIVO experiment; **d**–**f** MMBS simulation. **a**, **d** Axial contact force on the tibia (magnitude). **b**, **e** Displacement of the femur relative to the tibia in the anteroposterior direction. **c**, **f** Internal/external rotation of the tibia

A shift of the femoral implant component relative to the femur by ± 3 mm in the anteroposterior direction was applied. The resulting profiles of the tibiofemoral contact force, the anteroposterior translation of the femur relative to the tibia, and the tibial internal–external rotation are presented in Fig. 6.

An anteroposterior shift of the femoral implant component considerably influences the joint kinematics and dynamics. The axial tibiofemoral contact force remains on the same respective level across both test series (VIVO/MMBS) for small flexion angles up to approximately 5°. However, as flexion increases, the profiles diverge notably. With an anterior displacement of 3 mm, the contact force decreases relative to the reference configuration starting at 5° flexion, reaching a minimum of 101 N in the VIVO experiment and 67 N in the MMBS simulation at 80° flexion. These values represent reductions of 392 N (− 80%) and 386 N (− 85%), respectively, compared to the reference. Conversely, a posterior shift of the femoral implant component by 3 mm increases the contact force, reaching a maximum of 1300 N (VIVO experiment) and 1529 N (MMBS simulation) at 80° flexion. Compared to the reference, this represents an increase of 807 N (+ 164%) and 1076 N (+ 238%), respectively.

Differences are also evident in the anteroposterior displacement of the femur relative to the tibia. An anterior displacement of the femoral implant component by 3 mm results in an overall more posterior positioning of the femur. Compared to the reference configuration, the displacement ranges from about 1.4 mm at the start of flexion to a maximum of about 3 mm at around 70° flexion in both the VIVO experiment and the MMBS simulation. Notably, the characteristic inflection points in the profiles around 65° flexion, followed by a posterior movement of the femur, are absent in both cases. In contrast, a 3 mm posterior shift of the femoral implant component has a smaller effect. It causes a constant anterior shift of the femur by approximately 1 mm in both the experiment and simulation up to a flexion angle of about 60°. The onset of femoral rollback occurs about 5° earlier at around 60° flexion compared to the reference configuration. As a result, at maximum flexion angles, the femur is positioned more posterior by about 1 mm (VIVO experiment) and 2.3 mm (MMBS simulation).

Regarding tibial internal–external rotation, differences between the reference position and an anteroposterior shift of the femoral component are relatively small. In both the VIVO experiment and the MMBS simulation, an anterior displacement of the femoral component by 3 mm results in an approximately 1°–2° increase in tibial external rotation over most of the flexion range. With increasing flexion, starting around 60°, internal rotation occurs, particularly pronounced in the MMBS simulation. On the other hand, a posteriorly displaced femoral component has minimal effect on tibial rotation at low flexion angles up to 40°, and leads to a slight increase in external rotation of approximately 1° at higher flexion angles in both the experiment and the simulation.

### Mediolateral shift of the femoral implant component

**Fig. 7:**
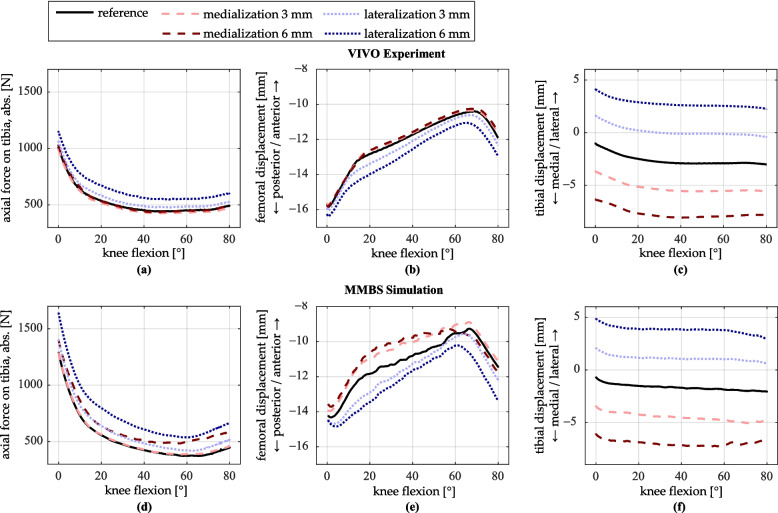
Simulation results for mediolateral shift of the femoral implant. **a**–**c** VIVO experiment; **d**–**f** MMBS simulation. **a**, **d** Axial contact force on the tibia (magnitude). **b**, **e** Displacement of the femur relative to the tibia in the anteroposterior direction. **c**, **f** Displacement of the tibia relative to the femur in the mediolateral direction

The femoral implant component was shifted in the mediolateral direction by ± 3 mm and ± 6 mm. Figure 7 shows the resulting tibiofemoral contact forces and the anteroposterior femur position. Since a mediolateral shift of the femoral implant is also expected to have a notable effect on the tibial displacement in the same direction, the mediolateral position of the tibia is additionally shown.

Medial shift of the femoral implant component has only minor effects on the contact force in the VIVO experiment. In contrast, the MMBS simulation shows an increase in contact force of up to 150 N with a 6 mm medial shift. The effects of lateral shift are more pronounced in both cases: a 6 mm lateral shift leads to an increase in contact force of approximately 100 N in the experiment and about 200 N in the simulation. A 3 mm lateral shift results in a smaller increase in contact force for both cases.

In the VIVO experiment, medial shift of the femoral implant component has no considerable effect on the anteroposterior position of the femur. In contrast, the MMBS simulation shows the femur positioned approximately 1 mm more anteriorly for both levels of medialization up to 60° of flexion. With a 6 mm lateral shift, both the experiment and simulation show an overall approximately 0.5 mm more posterior femur position; with a lateral shift of 3 mm, this trend is also observable to a lesser extent.

The mediolateral tibial position profiles run approximately parallel across all levels of femoral implant component shift. A medial or lateral shift of 3 mm or 6 mm of the femoral implant results in a corresponding tibial displacement in the same direction by approximately 2.5 mm or 5 mm, respectively, in both the experiment and the simulation.

### Internal/external twist of the tibial insert

**Fig. 8:**
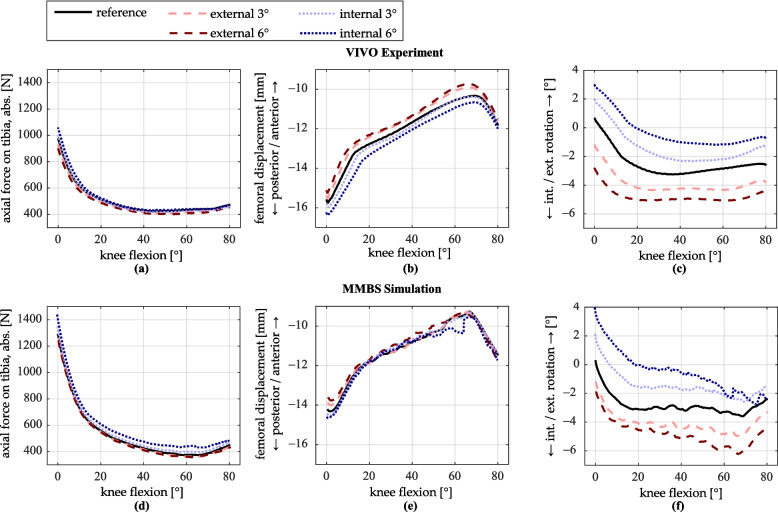
Simulation results for internal/external twist of the tibial insert. **a**–**c** VIVO experiment; **d**–**f** MMBS simulation. **a**, **d** Axial contact force on the tibia (magnitude). **b**, **e** Displacement of the femur relative to the tibia in the anteroposterior direction. **c**, **f** Internal/external rotation of the tibia

The tibial insert was twisted by ± 3° and ± 6° around the tibial mechanical axis. Figure 8 presents the tibiofemoral contact force, the femoral displacement, and the tibial internal–external rotation.

The influence of internal–external twist of the tibial insert on the tibiofemoral contact force is generally minor in both the experiment and the simulation. The largest difference is an increase of approximately 70 N for the insert rotated 6° internally in the MMBS simulation.

In the VIVO experiment, both levels of external twist of the tibial insert result in the femur being positioned approximately 0.5 mm more anteriorly. In contrast, a 6° internal twist of the insert leads to a femoral position that is approximately 0.5 mm more posterior. In the simulation, the differences in anteroposterior femur position are smaller across all levels of insert twist.

Regarding tibial internal–external rotation, the overall patterns remain similar in shape. However, the tibia consistently rotates in the opposite direction of the insert twist, with deviations ranging between 0.5° and 1.5°.

### Variation of the tibial insert thickness

**Fig. 9:**
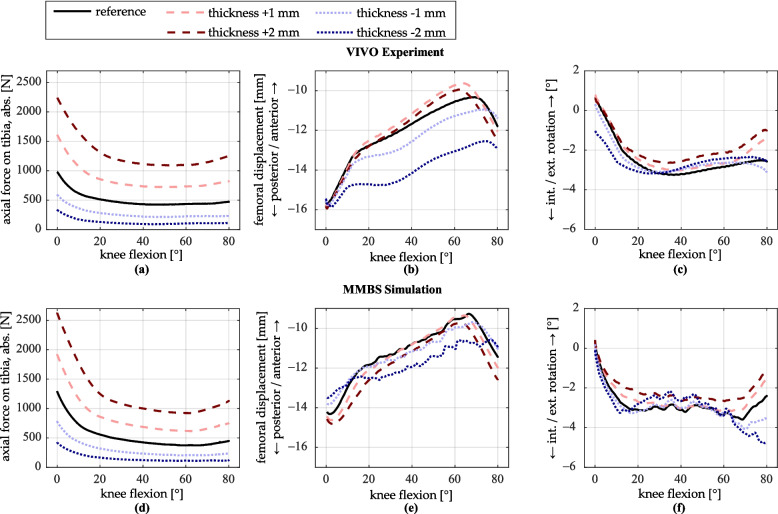
Simulation results for variation of the tibial insert thickness. **a**–**c** VIVO experiment; **d**–**f** MMBS simulation. **a**, **d** Axial contact force on the tibia (magnitude). **b**, **e** Displacement of the femur relative to the tibia in the anteroposterior direction. **c**, **f** Internal/external rotation of the tibia

The thickness of the tibial insert was varied by ± 1 mm and ± 2 mm. Figure 9 presents the resulting profiles of tibiofemoral contact force, the anteroposterior position of the femur relative to the tibia, and the tibial rotation.

Varying the thickness of the tibial insert has a considerable effect on the axial tibiofemoral contact force. An increase of 2 mm in insert thickness raises the contact force at 0° flexion by 1263 N to 2240 N (+ 129%) in the VIVO experiment, and by 1337 N to 2631 N (+ 103%) in the MMBS simulation, compared to the reference. As flexion increases, the force difference decreases to approximately + 600 N (experiment) and + 550 N (simulation). Reducing the insert thickness by 2 mm leads to a maximum force reduction at 0° flexion of 646 N (− 66%) in the experiment and 872 N (− 67%) in the simulation. With increasing flexion, the force is still about 300 N lower in both the experiment and the simulation. The force profiles for a ± 1 mm thickness adjustment lie approximately midway between the reference and the ± 2 mm conditions.

In the VIVO experiment, an increase in insert thickness results in a slightly more anterior femoral position relative to the tibia from approximately 15° flexion onward, reaching a maximum difference of about 0.5 mm at 60° of flexion. This behavior is not observed in the MMBS simulation, where the femur is located slightly more posterior at the beginning of flexion but converges towards the reference position as flexion increases. In both setups, the onset of the femoral posterior rollback occurs earlier: for a 2 mm increase in insert thickness, about 5° earlier at 60° flexion. Conversely, reducing the insert thickness causes a generally more posterior femoral position in both test environments. This effect is more pronounced in the VIVO experiment, with a maximum deviation of about 2.5 mm from the reference, compared to approximately 1.2 mm in the simulation for a 2 mm insert height reduction. In both setups, femoral rollback begins roughly 10° of flexion later compared to the reference, and the overall extent of posterior movement is reduced.

Up to 60° of flexion, the effects of differing insert thicknesses on tibial internal–external rotation are relatively small, with maximal deviations of approximately 1° compared to the reference configuration. Beyond 60° of flexion, increasing the insert thickness results in greater external tibial rotation, whereas decreasing the insert thickness leads to greater internal rotation, which is particularly evident in the MMBS simulation. Overall, the curves show good agreement between the VIVO experiment and the MMBS simulation.

## Discussion

### Anteroposterior shift of the femoral implant component

Due to the structural compliance of the VIVO joint simulator’s actuators in combination with the initial contact force, the contact force in the VIVO experiment is lower than the numerically determined force in the MMBS simulation at high load levels, and higher at low load levels. This effect has been thoroughly investigated in a previous study [43] and will not be discussed further here. Nevertheless, the overall force patterns and the relative percentage changes are comparable between the experiment and the simulation.

A clear influence of the femoral implant component’s anteroposterior position on tibiofemoral contact force is observed with increasing flexion. When the femoral implant is positioned more posteriorly, the radius of curvature of the component increases as flexion progresses, resulting in greater separation between the bones and consequently higher ligament elongations and forces. Conversely, an anterior shift of the femoral implant reverses this effect. For small flexion angles, the influence of the anteroposterior femur position on the joint force is minimal, which is confirmed by the literature [15].

**Fig. 10:**
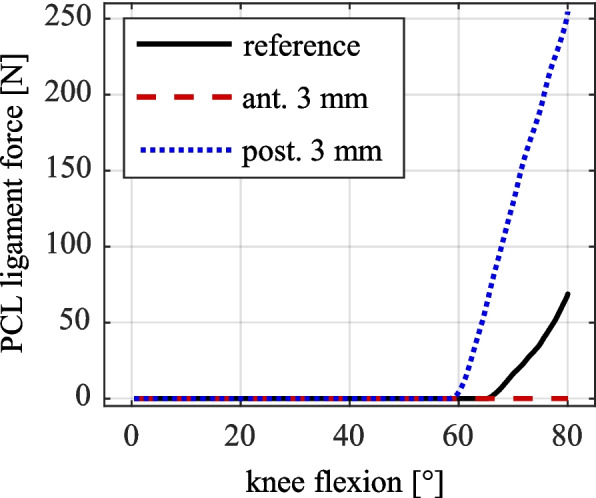
PCL ligament force (sum of both ligament bundles) for reference, anterior and posterior shift of femoral implant component

It is therefore expected that the force progression of the PCL, which is tensioned primarily at high flexion angles, is particularly affected by the anteroposterior shift of the femoral implant component. To confirm this assumption, the ligament forces of the PCL for the different displacement stages of the femoral implant are evaluated using the MMBS model and visualized in Fig. 10 as the respective sums of the two modeled bundles.

As can be seen in Fig. 10, a posterior shift of the femoral implant leads to a considerable increase in PCL force by approximately 270% to 253 N, while an anterior shift results in a complete absence of PCL tension for the present parametrization of ligaments.

A generally good agreement was observed between the kinematic results of the VIVO experiment and the MMBS simulation. For both femoral anteroposterior position and tibial rotation, all curves show similar shapes between the two datasets. Differences resulting from an anteroposterior shift of the femoral implant are represented by changes of comparable magnitude and direction. This indicates that the presented method is suitable for simulating the effects of implant shifts on kinematics in the VIVO simulator.

While an anteroposterior shift of the femoral implant has only a small effect on the tibial internal–external rotation, its impact on the femur’s anteroposterior position relative to the tibia is more pronounced. This is especially evident in the case of an anterior shift of the femoral implant component. The resulting more posterior femur position reflects the new equilibrium state established by the altered implant position. The absence of femoral rollback in this case was also reported by Kebbach [26] and can be attributed to the lack of PCL tension, as shown in Fig. 10. In contrast, posterior shift of the femoral implant leads to earlier and more pronounced femoral rollback, driven by an earlier and overall greater PCL tension.

### Mediolateral shift of the femoral implant component

**Fig. 11:**
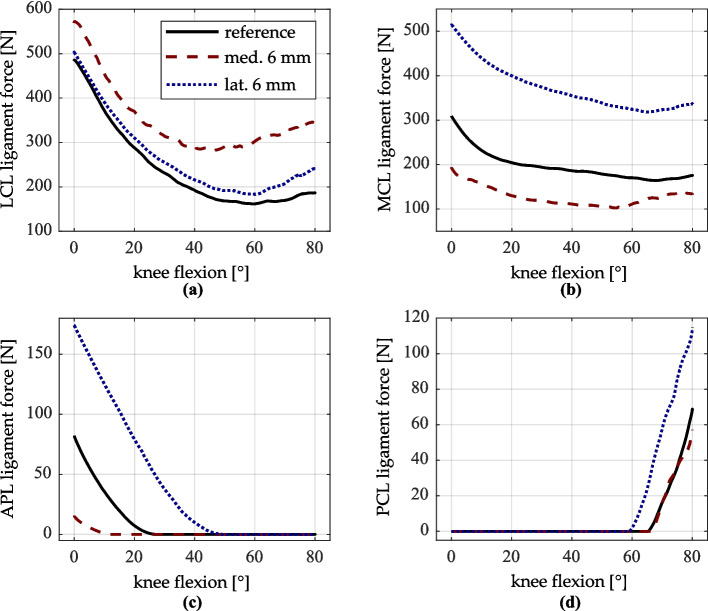
LCL, MCL, APL, and PCL ligament forces (respective sums of all ligament bundles) for reference, medial and lateral shift of femoral implant component. **a** LCL ligament force. **b** MCL ligament force. **c** APL ligament force. **d** PCL ligament force

When comparing the contact forces between the VIVO experiment and the MMBS simulation, the force curve profiles and the relative percentage changes show clear similarities. An increase in contact force resulting in a mediolateral shift of the femoral implant component indicates a tighter ligamentous structure in the joint. To confirm this interpretation, Fig. 11 shows the summed force curves for selected ligaments (LCL, MCL, APL, PCL) for mediolateral shifts of the femoral implant by ± 6 mm.

A lateral shift of the femoral implant component by 6 mm results in an average increase of approximately 70% in MCL bundle forces during extension (Fig. 11b), while the LCL bundle forces also increase slightly (Fig. 11a). Conversely, medial shift of the femoral implant leads to increased LCL forces and decreased MCL forces. These trends are consistent with findings by Cheng et al. [15], who reported increased loading in the lateral condyle following medialization, and in the medial condyle following lateralization of the femoral implant. However, the lack of a corresponding increase in contact force with medial implant shift in the VIVO experiment contradicts both the simulation and the literature. It may be attributed to inaccuracies in the mediolateral positioning of the tibial insert in the reference configuration.

Regarding the anteroposterior position of the femur, a greater influence can be observed with the lateral shift of the femoral implant component in the VIVO experiment, which leads to an overall more posterior femur position. The reasons for this kinematic change are difficult to determine, but differences in the tension of the APL and PCL may contribute. The APL originates at the proximal head of the fibula and runs obliquely along the posterior joint capsule in a medial and proximal direction, merging with the OPL (Fig. 1d). Its anatomical course makes it sensitive to mediolateral kinematic changes. As shown in Fig. 11c, the lateral shift of the femoral implant leads to an increase in APL force from 77 N to 172 N in full extension, while the medial shift reduces APL force to 18 N. Increased APL tension on the posterior side of the joint can thus be given as a reason for a more posterior femur position at low flexion angles. Conversely, reduced APL tension allows for greater anterior translation of the femur relative to the tibia, which can be observed particularly in the MMBS simulation (Fig. 7e).

Similarly, PCL tension increases with lateral shift from 68 N to 117 N at 80° of flexion. The decrease with medial shift is less prominent (Fig. 11d). At higher flexion angles, increased PCL tension plausibly explains a more posterior femur position, as the PCL is primarily responsible for the femoral posterior rollback during flexion [43, 46, 47]. While a direct correlation between APL/PCL tension and anteroposterior femoral positioning due to mediolateral femoral prosthesis shift has not yet been described in the literature, increased femoral rollback with lateral femoral implant component shift has previously been reported [23].

The nearly parallel mediolateral displacements of the tibia in both the experiment and simulation (Fig. 6c and 6f) directly result from the mediolateral shift of the femoral implant, establishing a new equilibrium configuration.

### Internal/external twist of the tibial insert

The minor influence of insert twist on tibiofemoral contact force suggests only small differences in the tension of ligaments. The literature presents varying findings regarding ligament tensions for different insert twists. For example, Kuriyama et al. [19] found that an internally twisted insert increases tension in the collateral ligaments, whereas Fottner et al. [17] observed the same effect with an externally twisted insert. Therefore, the overall minor influence on tibiofemoral contact force observed in this study appears plausible.

Slight changes in anteroposterior femur position are supported by previous research: Steinbrück et al. [22] reported a femur position approximately 1 mm more posterior with a 3° internally twisted insert, and to a lesser extent, a more anterior position with the same degree of external insert twist. Fottner et al. [17] also reported changes of similar magnitude.

The more pronounced changes in tibial internal–external rotation of the entire tibia can be attributed to the consequences of twisting the insert in the opposite direction, which leads to a new equilibrium configuration. This observation is consistent with findings from previous studies [17, 19, 22].

### Variation of the tibial insert thickness

**Fig. 12:**
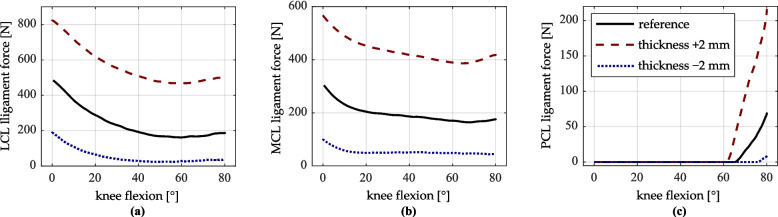
LCL, MCL, and PCL ligament forces (respective sums of all ligament bundles) for reference, increase, and decrease of tibial implant thickness. **a** LCL ligament force. **b** MCL ligament force. **c** PCL ligament force

Considerable changes in tibiofemoral contact force occur with variation in insert thickness. Increased insert thickness results in higher force levels due to a stiffer ligamentous apparatus, while decreased thickness produces lower forces due to increased joint laxity. As an example, Fig. 12 presents the summed force curves of the LCL, MCL, and PCL bundles for a variation in insert thickness of ± 2 mm.

Figure 12a and 12b show that an increase in insert thickness leads to a rise in LCL force ranging from 310 to 335 N, and in MCL force from 220 to 260 N. A decrease in insert thickness by 2 mm reduces the LCL force by 130 N to 300 N, and the MCL force by 117 N to 204 N. The PCL force, which is only under tension at high flexion angles, increases by up to 153 N and decreases by up to 62 N (Fig. 12c). The force increase associated with a thicker insert is greater than the force decrease with a thinner insert. This is attributed to the exponential stress–strain behavior of ligaments up to a ligament strain of 6% (Eq. 1).

Other studies [24, 26, 27] confirm the trend of increased or decreased individual ligament forces and contact force with changes in insert thickness. However, the magnitude of force changes for the same insert height variations reported in these studies is lower than that observed in the present study. These discrepancies may be due to differences in ligament parameterization, which is not always explicitly described in the literature. Additionally, the examined loading conditions differ; for example, Kebbach [26] investigated an active squat.

A more posterior femur position observed particularly in the VIVO experiment with reduced insert thickness can be attributed to the influence of the patella in combination with an overall looser ligamentous structure. Under quadriceps loading, the patella affects the anteroposterior femur position since it pushes the femur further posteriorly. This effect is amplified when the ligamentous structures are looser, as the posterior force exerted by the patella on the femur is opposed by lower ligament forces. Effects on the tibial rotation are relatively small and represented in a similar manner in both the VIVO experiment and the MMBS Simulation.

### Study limitations

The described methods for varying implant positions and overall testing using the VIVO joint simulator have some limitations worth noting. First, the insertion areas of the physically present artificial quadriceps muscle and the PL cannot be moved. Consequently, the forces applied through quadriceps loading act at the same locations as in the unchanged reference model. However, given the comparatively low and constant quadriceps force (40 N), no considerable influence is expected from this constraint.

Another limitation of the described setup lies in the placement of the force-moment sensor integrated into the distal actuator, which is located below the tibial attachment point of the PL. Therefore, the additional joint compressive force caused by loading the M. quadriceps femoris is not captured. The exact joint compression force could be measured using additional sensors, such as pressure-sensitive film between the contact surfaces [48, 49].

Over prolonged use of the experimental setup, material fatigue was observed in the steel wire near the deflection pulley, making a replacement necessary. The current arrangement was chosen so that the centrally positioned quadriceps wire does not have to be guided past the adduction-abduction arm of the simulator located above it (Fig. 3). To improve durability, alternative materials with a higher resistance to alternating stress (e.g., based on polyethylene fiber), or a different guidance path for the steel wire could be considered in future experiments.

A geometric measuring method was used to verify the position of the implant components using a coordinate measuring device. This required manually positioning the measuring probe as precisely as possible at predefined points, during which minor deviations cannot be ruled out.

Lastly, when comparing findings of the present study to results from the literature and the MMBS, it should be noted that the experimental outcomes are affected by the structural compliance of the VIVO joint simulator [43]. Additionally, the consideration of ligament wrapping is not feasible by means of the VIVO joint simulator’s virtual ligament model, while wrapping influences the dynamics of the artificial knee joint as it helps to ensure that non-physiological motions do not occur [50].

## Conclusions

Across all variations in implant positions that were carried out, good agreement was achieved between the VIVO experiment and the MMBS simulation, particularly with regard to the kinematic characteristics. The VIVO joint simulator plausibly reproduces the effects of implant malalignment, which is further supported by comparisons with findings from the literature. This demonstrates that adjusting the ligament insertion points is efficient for simulating implant shifts within the bone while maintaining the mounting of the implant components on the simulator. The method thus requires no modifications to the physical setup; only a recalculation of the ligament reference strains is necessary. In addition to the exemplary variations in implant positions that were presented, the method allows for theoretical shifts and twists in any direction and of any magnitude, offering a high degree of flexibility.

## Data Availability

The original contributions presented in the study are included in the article, further inquiries can be directed to the corresponding author.

## Abbreviations

APL: Arcuate popliteal ligament
DOF: Degree of freedom
ILC: Iterative learning control
LCL: Lateral collateral ligament
MCL: Medial collateral ligament
dMCL: Deep MCL
opMCL: Oblique posterior MCL
MMBS: Musculoskeletal multibody system
OPL: Oblique popliteal ligament
PCAP: Posterior capsule
PCL: Posterior cruciate ligament
PL: Patellar ligament
TKA: Total knee arthroplasty
TKR: Total knee replacement
